# Use of a Chimeric Hsp70 to Enhance the Quality of Recombinant *Plasmodium falciparum S*-Adenosylmethionine Decarboxylase Protein Produced in *Escherichia coli*

**DOI:** 10.1371/journal.pone.0152626

**Published:** 2016-03-31

**Authors:** Xolani Henry Makhoba, Adélle Burger, Dina Coertzen, Tawanda Zininga, Lyn-Marie Birkholtz, Addmore Shonhai

**Affiliations:** 1 Department of Biochemistry and Microbiology, University of Zululand, KwaDlangezwa, South Africa; 2 Department of Biochemistry, School of Mathematical & Natural Sciences, University of Venda, Thohoyandou, South Africa; 3 Department of Biochemistry, Centre for Sustainable Malaria Control, University of Pretoria, Pretoria, South Africa; National Centre for Cell Science, INDIA

## Abstract

*S*-adenosylmethionine decarboxylase (PfAdoMetDC) from *Plasmodium falciparum* is a prospective antimalarial drug target. The production of recombinant PfAdoMetDC for biochemical validation as a drug target is important. The production of PfAdoMetDC in *Escherichia coli* has been reported to result in unsatisfactory yields and poor quality product. The co-expression of recombinant proteins with molecular chaperones has been proposed as one way to improve the production of the former in *E*. *coli*. *E*. *coli* heat shock proteins DnaK, GroEL-GroES and DnaJ have previously been used to enhance production of some recombinant proteins. However, the outcomes were inconsistent. An Hsp70 chimeric protein, KPf, which is made up of the ATPase domain of *E*. *coli* DnaK and the substrate binding domain of *P*. *falciparum* Hsp70 (PfHsp70) has been previously shown to exhibit chaperone function when it was expressed in *E*. *coli* cells whose resident Hsp70 (DnaK) function was impaired. We proposed that because of its domain constitution, KPf would most likely be recognised by *E*. *coli* Hsp70 co-chaperones. Furthermore, because it possesses a substrate binding domain of plasmodial origin, KPf would be primed to recognise recombinant PfAdoMetDC expressed in *E*. *coli*. First, using site-directed mutagenesis, followed by complementation assays, we established that KPf with a mutation in the hydrophobic residue located in its substrate binding cavity was functionally compromised. We further co-expressed PfAdoMetDC with KPf, PfHsp70 and DnaK in *E*. *coli* cells either in the absence or presence of over-expressed GroEL-GroES chaperonin. The folded and functional status of the produced PfAdoMetDC was assessed using limited proteolysis and enzyme assays. PfAdoMetDC co-expressed with KPf and PfHsp70 exhibited improved activity compared to protein co-expressed with over-expressed DnaK. Our findings suggest that chimeric KPf may be an ideal Hsp70 co-expression partner for the production of recombinant plasmodial proteins in *E*. *coli*.

## Introduction

*E*. *coli* is often the host of choice in the production of recombinant proteins. However, one of the challenges of producing recombinant proteins in *E*. *coli* remains that the products are occasionally released from ribosomes as insoluble inclusion bodies. In addition, the use of strong promoters and high inducer concentrations can generate product yields exceeding 50% of the total cellular protein [1]. Under such circumstances, the rate of protein production overwhelms the protein folding machinery, resulting in the generation of poor quality, mis-folded recombinant proteins. Mehlin and co-workers [2] analysed 1000 genes from *P*. *falciparum* parasites that were over-expressed in *E*. *coli* and reported that only 337 were successfully produced. Of these, only 63 were reported as soluble proteins. It has been proposed that the recombinant expression of plasmodial proteins in *E*. *coli* in the presence of molecular chaperones of similar origin could improve both yield and quality of the product [3][4].

*S*-adenosylmethionine decarboxylase (PfAdoMetDC) of the malaria parasite, *P*. *falciparum*, is a component of the unique bifunctional PfAdoMetDC-ODC (*S*-adenosylmethionine decarboxylase-ornithine decarboxylase) controlling the biosynthesis of essential polyamines, making it a potential antimalarial drug target [5][6]. Obtaining a pure and active form of monofunctional PfAdoMetDC in fairly large quantities would facilitate its further characterisation by methods such as crystallisation. Although recombinant PfAdoMetDC has been expressed in *E*. *coli*, the protein co-purified with *E*. *coli* proteins, amongst them DnaK [7]. DnaK belongs to the heat shock protein 70 (Hsp70) family of molecular chaperones whose main function is to bind mis-folded proteins to allow them to fold. It is therefore plausible that PfAdoMetDC is released from ribosomes in mis-folded status, attracting DnaK. Hsp70/DnaK binds proteins exhibiting extended hydrophobic patches which would normally be buried in a fully folded protein [8][9]. The binding of DnaK to mis-folded proteins facilitates their refolding [8][9].

Heat shock proteins (Hsps) constitute the central molecular machinery of the cell which facilitates protein folding. Hsp70/DnaK is one of the most prominent molecular chaperones. Hsp40 and GrpE co-operate with Hsp70 in chaperone action. The role of Hsp40 (*E*. *coli* DnaJ) is to bind substrates and present them to Hsp70 and simultaneously modulate the ATPase activity of Hsp70 [10]. Hsp40s thus regulate the functional specificity of Hsp70. In the ADP-bound state, Hsp70 binds to its substrates with high affinity, whilst it releases its substrates in the ATP-bound state [11]. The nucleotide exchange function of DnaK is facilitated by a co-chaperone named GrpE [12].

An Hsp70 from *P*. *falciparum* parasites (PfHsp70), which is thought to be important for quality control in the parasite, was previously over-expressed [13] in *E*. *coli dnaK756* cells whose DnaK is functionally compromised [14]. In light of its capability to exhibit chaperone function in *E*. *coli* cells, PfHsp70 was previously co-expressed with *P*. *falciparum* GTP cyclohydrolase I (PfGCHI) in *E*. *coli* [4]. It was reported that the co-expression of PfGCH1 with PfHsp70 led to improved quality of the PfGCH1[4].

**Fig 1:**
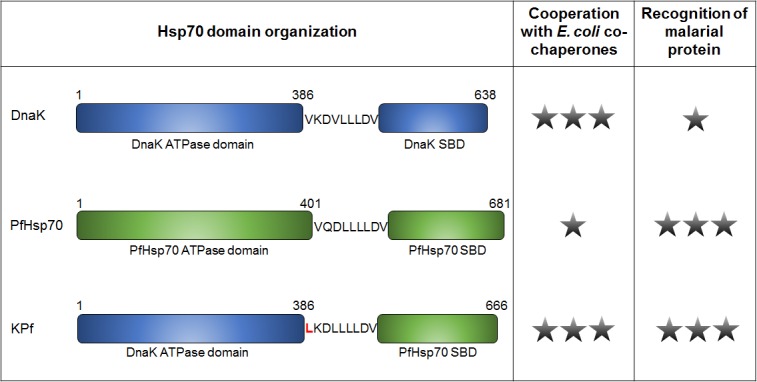
Possible cooperation of KPf with *E*. *coli* co-chaperones and capability to recognise recombinant malarial proteins. Schematic representation of the domain organisation of *E*. *coli* DnaK, PfHsp70 and their chimeric derivative, KPf. KPf is comprised of the ATPase domain of DnaK and the PfHsp70 substrate binding domain (SBD), connected by the linker. A single star represents a weak likelihood of cooperation with *E*. *coli* co-chaperones, or malarial protein recognition by the respective Hsp70 protein, and three stars represents a strong likelihood. This model proposes KPf as an ideal Hsp70 co-expression partner for production of malarial proteins in a bacterial host as it is likely to cooperate with *E*. *coli* co-chaperones as well as recognise substrates of malarial origin.

A chimeric Hsp70 protein, KPf, has previously been described (Fig 1) [13]. This chimeric protein was constructed by fusing the ATPase domain of *E*. *coli* DnaK to the substrate binding domain of PfHsp70 [13]. The over-expression of KPf led to protection of *E*. *coli dnaK756* cells (express a resident DnaK that is functionally compromised) against heat stress [13]. We surmised that KPf could serve as a more effective molecular chaperone partner for boosting the yield and quality of recombinant plasmodial proteins in *E*. *coli*. This is because it is likely to co-operate with *E*. *coli* co-chaperones (Fig 1). Furthermore, because it possesses the PfHsp70 substrate binding domain, it is likely to recognise target plasmodial recombinant proteins, facilitating their fold in *E*. *coli*.

GroEL, a protein that belongs to the Hsp60 family and is a barrel-shaped chaperonin whose structure is composed of 14 identical domains that make up seven distinct subunits [15]. *E*. *coli* GroEL is composed of an ATPase domain, a middle hinge-domain and an apical substrate binding domain. GroEL has a preference for substrates that range between 20–50 kDa and which are characterised by elaborate α/β or β + β topologies [16]. GroES is made up of a heptameric ring constituted by 10 kDa subunits which bind to the ends of the GroEL barrel and thus serving as the ‘lid’ of the GroEL barrel [17]. Production of supplemented GroEL-GroES has been associated with improved processing of recombinant protein produced in *E*. *coli* [18].

It has been proposed that although DnaK and trigger factor, another *E*. *coli* chaperone that facilitates folding of newly synthesised, both improve the folding process of newly synthesised peptides, they also significantly slow down the folding process [19]. It is possibly for this reason that co-expression of certain recombinant proteins with supplemented *E*. *coli* chaperones does not always yield positive outcomes. For instance, de Marco and co-workers [20] reported that only 26 of the 50 target proteins that were co-overproduced with supplemented *E*. *coli* chaperones showed enhanced solubility and improved yields. Altogether, this suggests that *E*. *coli* chaperones may not be acquiescent to facilitate folding of certain recombinant proteins, especially those of eukaryotic origin. For this reason, it has been proposed that rehosting the *E*. *coli* protein folding landscape by matching chaperones and target proteins from the same species for co-expression in *E*. *coli* could improve yields and quality of recombinant proteins [3]. Since production of malarial proteins in *E*. *coli* is problematic, this approach may possibly assist. Indeed, a previous study demonstrated that the co-expression of PfHsp70 with another plasmodial protein, PfGCHI, improved the solubility and functional status of the latter [4]. In the current study we investigated the merit of the chimeric Hsp70, KPf, as a chaperone co-expression partner towards improving the quality of recombinant PfAdoMetDC produced in *E*. *coli*. Our study further investigated the effect of co-expressing PfAdoMetDC with combinations of supplemented chaperones from both *E*. *coli* and *P*. *falciparum*. Our findings indicate that supplementation of chaperones of plasmodial origin improves the quality and stability of recombinant malarial proteins produced in *E*. *coli*. We discuss our findings with respect to their application in recombinant protein biotechnology and their impact on our understanding of protein folding in *E*. *coli*.

## Results

### Alteration of the hydrophobic pocket residue of KPf abrogates its chaperone function

**Fig 2:**
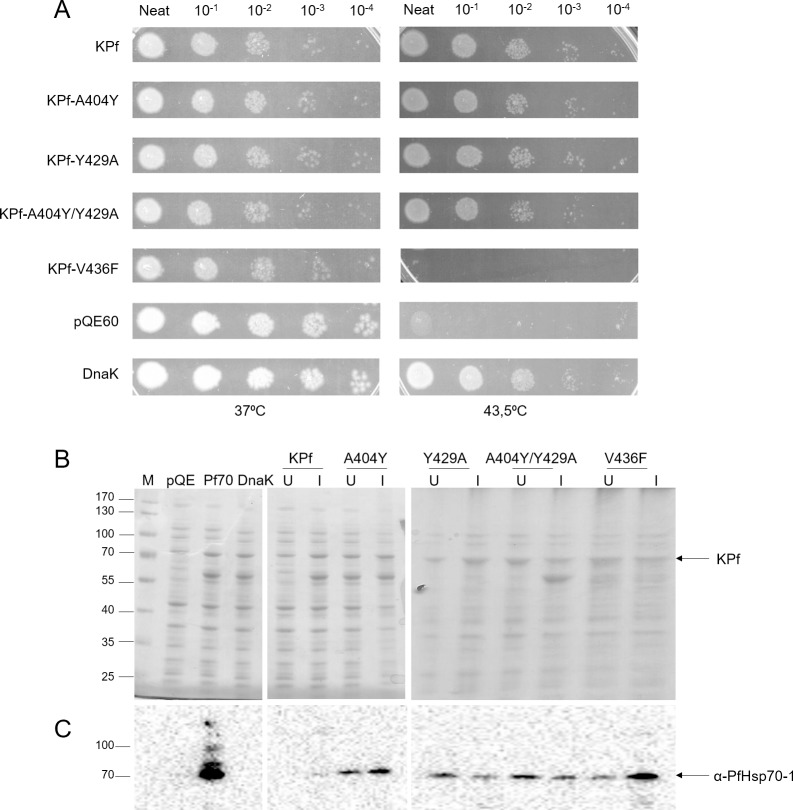
The hydrophobic pocket mutation abrogates the function of KPf. (A) *E*. *coli dnaK756* cells transformed with plasmid constructs expressing KPf and its substrate binding cavity mutants were incubated at 37°C and 43.5°C, respectively. The negative control consisted of cells transformed with pQE60 plasmid vector whilst the positive control was represented by cells transformed with the pQE60/DnaK plasmid. (B) SDS-PAGE and Western analyses for the exogenous expression of KPf and the respective substrate binding cavity derivatives in *E*. *coli dnaK756* cells. Cells transformed with plasmid vector (pQE60) were used as negative control. The labels on the top of the gel panels represent the different proteins that were expressed as well as the vector control (pQE60). In each case, the left and right hand side lanes represents the sample that was taken before induction and 5 hours after induction with 1 mM IPTG, respectively. Numbers on the left handside represent protein markers (Fermentas) in kDa.

Hsp70 binds substrates through its substrate binding cavity characterised by three components: the α-helical lid, the arch defined by residues A429 and M404 and the hydrophobic central pocket composed of the V436 residue, in prokaryotes [21]. The substrate binding arch of DnaK is constituted by residues M404 and A429; and in Hsp70s of eukaryotic origin, the residues in these positions are A and Y, respectively [22]. Hence Hsp70s of eukaryotic origin are regarded as possessing an ‘inverted’ arch compared to that of DnaK [23]. The arch is thought to make direct contact with substrates, allowing access to acidic and hydrophobic enriched peptides [23]. Apart from the arch residues, another important component regulating interaction of Hsp70 with its substrates is a highly conserved valine residue (V436 in DnaK) that is located in the substrate binding cavity of Hsp70 [21]. KPf was previously shown to protect *E*. *coli dnaK756* cells against heat stress [13]. Since KPf is a chimeric Hsp70 which was made up by combining the ATPase domain of DnaK and the substrate binding domain of PfHsp70; we surmised that it possessed two key advantages over DnaK and PfHsp70 as co-expression chaperone in recombinant protein production: (1) it possibly interacts with DnaJ and (2) its substrate binding domain is primed to bind malarial proteins. We investigated if making changes in the arch and hydrophobic pocket of KPf would influence its function in cytoprotecting *E*. *coli dnaK756* cells against heat stress. We made the following substitutions to investigate if these would abrogate KPf function: A404Y; Y429A; A436F and A404Y/Y429A. The substitutions introduced in the substrate binding cavity of KPf (A404Y, Y429A and A404Y/Y429A) did not influence its function (Fig 2A). Only the V436F substitution in the hydrophobic pocket of KPf led to a functional abrogation (Fig 2A). SDS-PAGE analysis showed that the protein was produced to a level comparable to the original KPf chimera (Fig 2B). This suggests that the V to F substitution led to blockage of the hydrophobic pocket, restricting access of substrates to the substrate binding cavity of KPf.

### Co-expression of PfAdoMetDC with supplemented molecular chaperones

**Fig 3:**
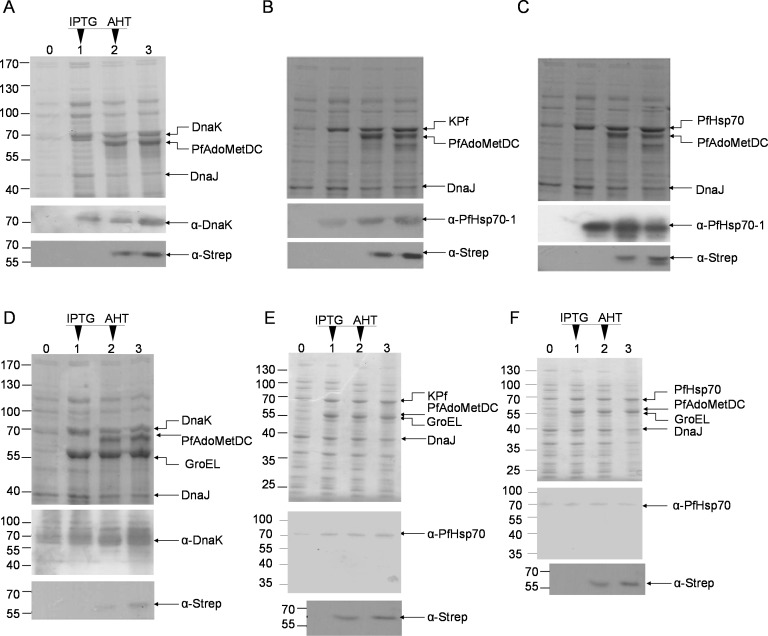
Co-expression of PfAdoMetDC with various chaperone sets in *E*. *coli* BL21 (DE3) Star cells. Whole cell lysates from the co-expression of PfAdoMetDC with chaperone sets (A) DnaK + DnaJ, (B) KPf + DnaJ, (C) PfHsp70 + DnaJ, (D) DnaK + GroEL + DnaJ, (E) KPf + GroEL + DnaJ, and (F) PfHsp70 + GroEL + DnaJ were analysed on SDS-PAGE (upper panel) and confirmed by Western blot analysis (lower panels). Lanes U–uninduced whole cell lysate; I–whole cell lysate 1 hour post IPTG (1 mM) induction; A1 –whole cell lysate 1 hour post AHT (2 ng/ml) induction; A2 –whole cell lysate 2 hours post AHT plus IPTG induction. Lower panels: DnaK was detected using α-DnaK antibody, PfHsp70 was detected using α-PfHsp70 antibody and PfAdoMetDC (60 kDa) was detected using α-Strep antibody. Numbers to the left represent protein markers (Fermentas) in kDa.

Upon induction using AHT, PfAdoMetDC was recombinantly expressed in *E*. *coli* BL21 (DE3) Star cells (Fig 3). Additionally, the following chaperones could be successfully co-expressed with PfAdoMetDC: DnaK+DnaJ, DnaK+DnaJ+KPf and DnaK+DnaJ+PfHsp70. (Fig 3A–3C). Supplemented GroEL-GroES was also successfully expressed in tandem with one of the following chaperone sets: DnaK+DnaJ; KPf+DnaJ or PfHsp70+DnaJ (Fig 3D–3F). The successful co-expression of PfAdoMetDC with the above-mentioned chaperone sets created a platform for further enquiries regarding their role in influencing the quality of PfAdoMetDC. We noted that PfHsp70 and KPf co-expressed with supplemented GroEL-GroES were resolved on Western blots as full length protein forms and products of smaller sizes (Fig 2). It appears that GroEL-GroES overproduction may have compromised the processing of PfHsp70 and KPf as full length proteins in *E*. *coli* BL21 (DE3) Star cells. Nonetheless, a fair amount of full length forms of the two chaperones that were produced in the presence of supplemented GroEL-GroES.

There was no evidence that exogenous expression of DnaJ improved the levels of protein beyond those of the resident form of the protein (Fig 3A). Hsp40 proteins are generally produced at low levels *in vivo*, and over-expression of Hsp40 can, in certain cases lead to toxicity and a decrease in cell viability [24]. *E*. *coli* cells circumvent production of toxic levels of DnaK, GrpE and DnaJ by using these proteins as negative regulators of the expression of heat shock genes through their effect on the stability of σ^32^ [25]. Therefore, the over-expressed exogenous DnaJ may have suppressed the production of the resident form of the protein. Because GroEL and PfAdoMetDC are nearly of the same size (~60 kDa), their expression could not be resolved by SDS-PAGE (Fig 3, upper panels). To validate the expression of PfAdoMetDC we conducted Western blotting using α-Strep antibodies (Fig 3D–3F, lower panels). The expression of GroEL was confirmed by Western blot analysis (data not shown) that was conducted using α-Hsp60 antibodies [26].

### PfAdoMetDC co-expressed with supplemented plasmodial Hsp70s and GroEL/ES is associated with less contaminating species

**Fig 4:**
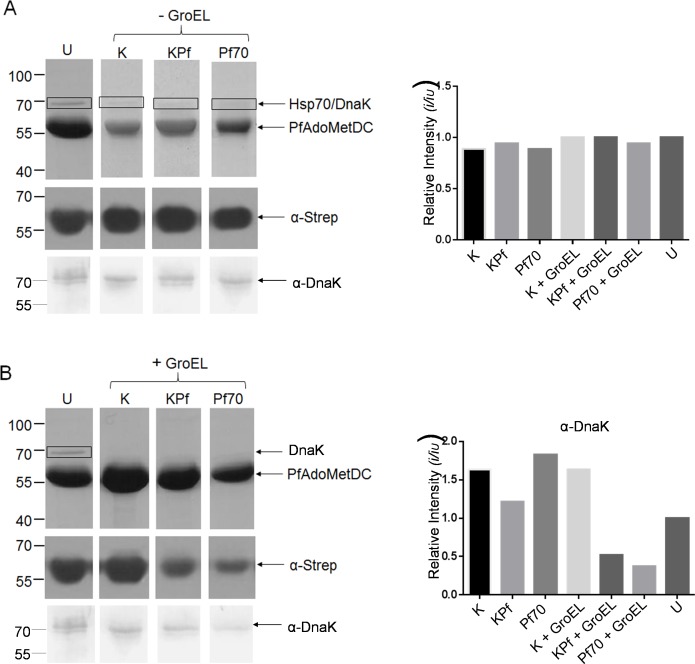
Co-expression of PfAdoMetDC with plasmodial Hsp70s and supplementary GroEL/ES improves quality of product. SDS-PAGE (top panel) and Western blot (lower panel) representing the purification of PfAdoMetDC expressed in *E*. *coli* BL21 (DE3) Star cells rehosted with various chaperone combinations. Lanes: **U**–PfAdoMetDC expressed in the absence of supplemented chaperones; K–PfAdoMetDC co-expressed with supplemented DnaK; KPf–PfAdoMetDC expressed in cells supplemented with KPf; Pf70 –PfAdoMetDC expressed in cells supplemented with PfHsp70; K-EL–PfAdoMetDC expressed in cells supplemented with DnaK and GroEL-GroES; KP-EL–PfAdoMetDC expressed in cells supplemented with KPf and GroEL-GroES; Pf70-EL–PfAdoMetDC expressed in cells supplemented with PfHsp70 and GroEL-GroES. Lower panels: Western blot analysis of PfAdoMetDC (60 kDa) and DnaK (70 kDa) detected using α-Strep and α-DnaK antibodies, respectively. Numbers to the left represent protein markers (Fermentas) in kDa. Densitometric analysis for the Western blots probed with α-Strep (C) and α-DnaK (D) antibodies, respectively. Relative intensities were compared to the sample U (representing PfAdoMetDC expression in the absence of supplemented chaperones). Band intensities were determined using Image J (http://imagej.nih.gov/ij/).

As in previous attempts to purify the protein [7], PfAdoMetDC expressed in *E*. *coli* BL21 (DE3) Star cells endowed with resident levels of DnaK co-purified with DnaK. In the current study, we also observed that DnaK co-purified with PfAdoMetDC (Fig 4). Thus our findings indicate that supplementation of DnaK does not improve the purity of recombinant PfAdoMetDC. This could be because DnaK binds to PfAdoMetDC stably, suggesting that the PfAdoMetDC protein was produced as a mis-folded species. In addition, in the absence of supplemented GroEL/ES, there is no evidence that KPf or PfHsp70 co-expression reduced DnaK contamination in the PfAdoMetDC protein that was purified (Fig 4). However, the introduction of GroEL/ES combined with either KPf or PfHsp70 led to a reduction in the level of DnaK contamination (Fig 4). Furthermore, PfHsp70 and KPf did not co-purify with PfAdoMetDC expressed either in the absence or presence of supplemented GroEL/ES (S1 Fig). Overall, this suggests that DnaK contamination could not be reduced by supplementing GroEL/ES only. On the other hand, supplemented GroEL/ES did appear to reduce DnaK contamination in the presence of KPf and PfHsp70.

### *E*. *coli* *∆dnaK* cells are capable of over-expressing PfAdoMetDC

**Fig 5:**
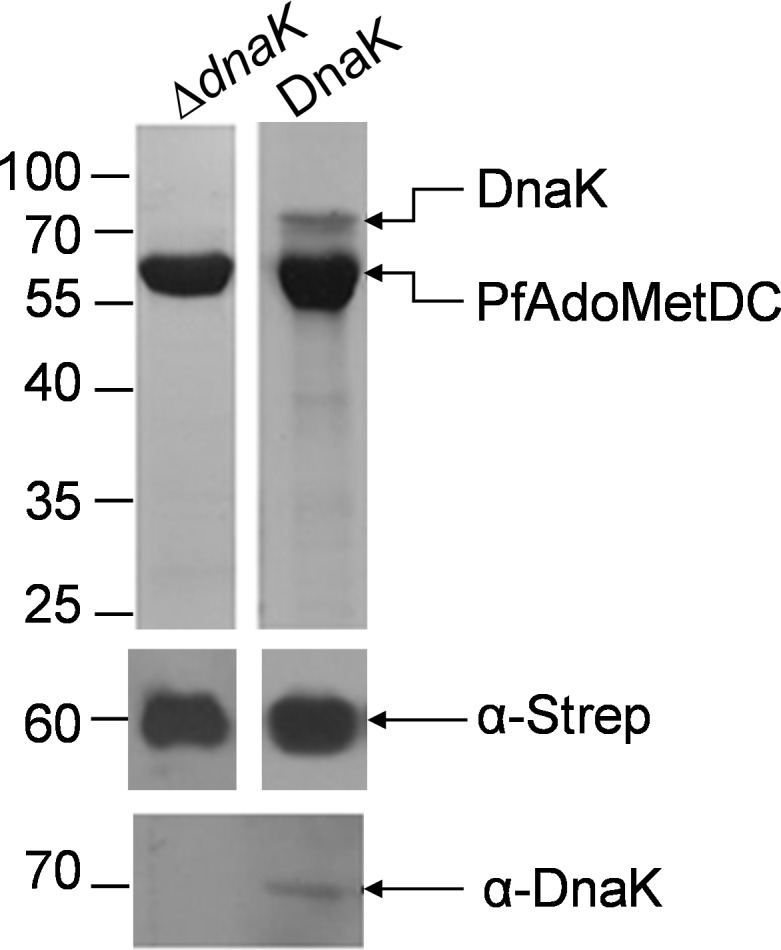
Purification of PfAdoMetDC produced in *E*. *coli ∆dnaK* cells. (A) SDS-PAGE (top panel) and Western (lower panels) representing the purification of PfAdoMetDC from *E*. *coli* Δ*dnaK* cells (lane Δ*dnaK*) and BL21 Star (DE3) cells (lane DnaK), respectively. PfAdoMetDC (60 kDa) and DnaK (70 kDa) were detected using α-Strep and α-DnaK antibodies, respectively. Numbers to the left represent protein markers (Fermentas) in kDa.

Although the supplementation of plasmodial chaperones (KPf and PfHsp70) along with over-expressed GroEL/ES reduced the levels of the persistent association of DnaK with the purified recombinant PfAdoMetDC, we enquired if *E*. *coli* cells deficient of DnaK would over-express PfAdoMetDC. In addition, we speculated that the co-expression of PfAdoMetDC in the presence of DnaK may confound the folding process of PfAdoMetDC. PfAdoMetDC was successfully expressed and purified from *E*. *coli* ∆*dnaK* cells (Fig 5). As expected, there was no contaminating DnaK in PfAdoMetDC purified from the *E*. *coli* ∆*dnaK* strain, as verified by Western blot analysis using α-DnaK antibody (Fig 5). The successful expression and purification of PfAdoMetDC from the *E*. *coli* ∆*dnaK* strain suggests that not all recombinant malarial proteins require DnaK for their production.

### PfAdoMetDC produced in *E*. *coli* cells rehosted with various chaperone constituents exhibits distinct structural features

**Fig 6:**
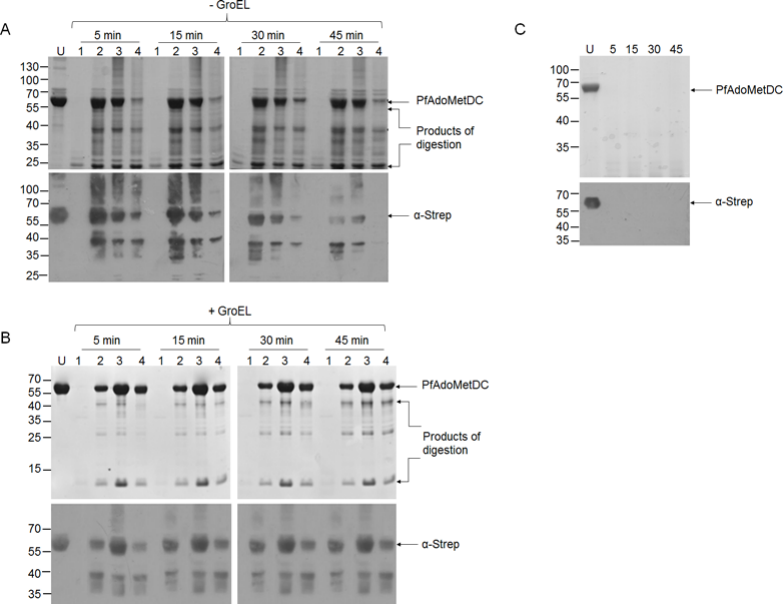
PfAdoMetDC co-expressed with various supplemented Hsp70 chaperones combined with GroEL-GroES exhibits variable proteolytic susceptibility. SDS-PAGE and accompanying Western blots for the resolution of PfAdoMetDC subjected to limited proteolysis (LP). (A) PfAdoMetDC co-expressed with Hsp70-DnaJ chaperone sets: The various lanes represent PfAdoMetDC expressed in the presence of the indicated chaperone sets: Lanes; U–undigested PfAdoMetDC; 1 –PfAdoMetDC expressed in cells that were not supplemented with over-expressed chaperones subjected to LP; 2 –PfAdoMetDC co-expressed with DnaK-DnaJ and to subjected to LP; 3 –PfAdoMetDC co-expressed with KPf-DnaJ and subjected to LP; 4 –PfAdoMetDC co-expressed with PfHsp70-DnaJ and subjected to LP. (B) PfAdoMetDC co-expressed with Hsp70-DnaJ-GroEL-GroES chaperone sets. Lanes; U–undigested PfAdoMetDC; 1 –PfAdoMetDC expressed in the absence of supplemented chaperones and subjected to LP; 2 –PfAdoMetDC co-expressed with DnaK-DnaJ-GroEL-GroES and subjected to LP; 3 –PfAdoMetDC co-expressed with KPf+DnaJ+GroEL-GroES and subjected to LP; 4 –PfAdoMetDC co-expressed with PfHsp70+DnaJ+GroEL-GroES and subjected to LP. Lower panels: Western blot analysis conducted using α-Strep antibody. (C) PfAdoMetDC expressed in *E*. *coli* ∆*dnaK* cells: Lanes; U—undigested PfAdoMetDC; the rest of the lanes contained PfAdoMetDC expressed and purified from *E*. *coli dnaK* minus cells and subjected to LP. Numbers to the left indicate protein marker (Fermentas) in kDa. The limited proteolysis by proteinase K was conducted using enzyme to substrate ratio of 1: 500. The duration of exposure to proteinase K is given in minutes.

We employed limited proteolysis to gain insight on the conformation of PfAdoMetDC expressed in the presence of the various Hsp70-DnaJ chaperones (Fig 6A). PfAdoMetDC produced in *E*. *coli* BL21 (DE3) Star cells, which were not supplemented with exogenous chaperones, was completely degraded by proteinase K within 5 minutes, generating small fragments (approximately 25 kDa in size) that could not be detected by Western blot analyses (Fig 6A, lane 1). PfAdoMetDC co-expressed with supplemented DnaK-DnaJ and KPf-DnaJ chaperones was fairly resistant to the action of proteinase K for 30 minutes (Fig 6A). Although the co-expression of PfAdoMetDC with PfHsp70-DnaJ improved the stability of the former, the protein was more susceptible to proteolytic action compared to protein recovered from cells that were supplemented with DnaK+DnaJ and KPf+DnaJ (Fig 6A). The products that were generated from PfAdoMetDC lysis exhibited unique profiles depending on the supplemented Hsp70 co-expression partner present (Fig 6A). This suggests that PfAdoMetDC produced in each case had a unique conformation. Overall, PfAdoMetDC expressed in *E*. *coli* cells supplemented with over-expressed DnaK+DnaJ was the most resistant to proteolytic action (Fig 6A).

PfAdoMetDC was further co-expressed with the following supplemented chaperone combinations: DnaK+DnaJ+GroEL-GroES, KPf+DnaJ+GroEL-GroES and PfHsp70+DnaJ+GroEL-GroES (Fig 6B). Recombinant PfAdoMetDC purified from *E*. *coli* BL21 (DE3) cells endowed with resident levels of DnaK and supplemented with GroEL-GroES (Fig 6B, lane 1) was just as susceptible to proteolytic digestion as the protein produced by cells expressing resident DnaK in the absence of supplemented GroEL-GroES (Fig 6A, lane 1). This suggests that GroEL-GroES was not able to improve the proteolytic stability of PfAdoMetDC produced in the presence of resident DnaK levels. However, of PfAdoMetDC produced by cells endowed with supplemented DnaK+DnaJ+GroEL-GroES had improved stability to proteolytic action (Fig 6B, lane 2). However, the product was not as stable as PfAdoMetDC produced by cells endowed with KPf+DnaJ+GroEL-GroES and PfHsp70+GroEL-GroES chaperone combinations (Fig 6B, lanes 3 and 4).

We further subjected PfAdoMetDC produced in the *E*. *coli* ∆*dnaK* strain to limited proteolysis. The protein was digested to smaller fragments, represented by faint bands on SDS-PAGE and which could not be resolved by Western blot analysis (Fig 6C). These findings demonstrate that recombinant PfAdoMetDC was produced in *E*. *coli* ∆*dnaK* cells as proteolytically susceptible molecule. PfAdoMetDC contains a Strep-tag at its C-terminus which was recognised by the α-Strep antibody we used [27]. We noted that some fragments that were generated upon proteolysis of PfAdoMetDC may have lost their Strep-tag as they were not detected by Western blotting in spite of their evident presence based on SDS-PAGE analysis.

### PfAdoMetDC produced in *E*. *coli* cells rehosted with Hsp70 chaperones exhibits improved enzymatic activity

**Fig 7:**
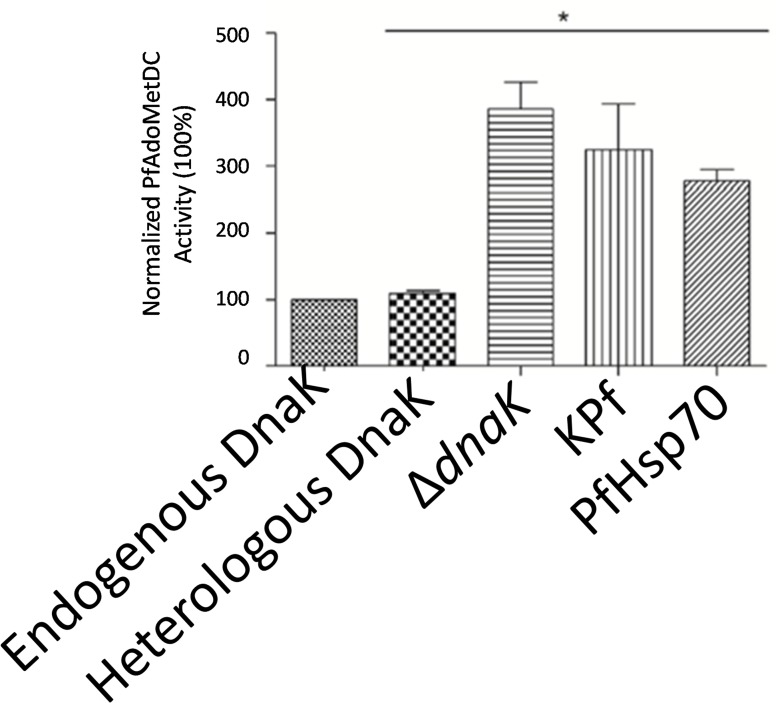
Enzymatic activity of purified recombinant PfAdoMetDC protein. The activities of PfAdoMetDC co-expressed with supplemented molecular chaperones—DnaK, KPf, and PfHsp70 were normalised against PfAdoMetDC that was produced in *E*. *coli* cells endowed with resident levels of molecular chaperones represented as “Resident DnaK”. “Over-expressed DnaK”, represents PfAdoMetDC that was co-produced with supplemented DnaK; and PfAdoMetDC that was purified from an *E*. *coli dnaK* minus strain is represented by “*∆dnaK”*. “Statistical significance was calculated using a Student’s t-test; * denotes p < 0.05.”.

The activity of PfAdoMetDC purified from *E*. *coli* under various chaperone supplementations was evaluated compared to the un-supplemented scenario (normalised to 100%; Fig 7). PfAdoMetDC co-expressed with supplemented DnaK did not exhibit higher activity than the protein produced in the presence of only resident DnaK levels. On the other hand, the activity of PfAdoMetDC, co-expressed with KPf and PfHsp70, was enhanced, resulting in a 3.24- and 2.77-fold increase, respectively (Fig 7). Unexpectedly, the activity of PfAdoMetDC produced the *E*. *coli* ∆*dnaK* strain exhibited the highest activity. It is interesting that although PfAdoMetDC produced in *E*. *coli* ∆*dnaK* cells was the least stable to proteolytic treatment, it exhibited the highest enzymatic activity.

On the other hand, PfAdoMetDC produced in cells that were supplemented with DnaK (in the absence of supplemented GroEL) was fairly resistant to proteolytic cleavage, but exhibited marginally improved activity (Figs 6A and 7). This shows that DnaK did not necessarily improve the activity of PfAdoMetDC. On the other hand, PfAdoMetDC co-expressed with either KPf or PfHsp70 exhibited both improved activity and resistance to proteolytic cleavage (Figs 6 and 7). Altogether, the findings suggest that PfAdoMetDC may have been recognised by both PfHsp70 and KPf, resulting in it attaining proper fold compared to protein that was produced in unmodified *E*. *coli* BL21 (DE3) Star cells.

### Assessment of the activities of the various individual chaperones and their combinations *in vitro*

**Fig 8:**
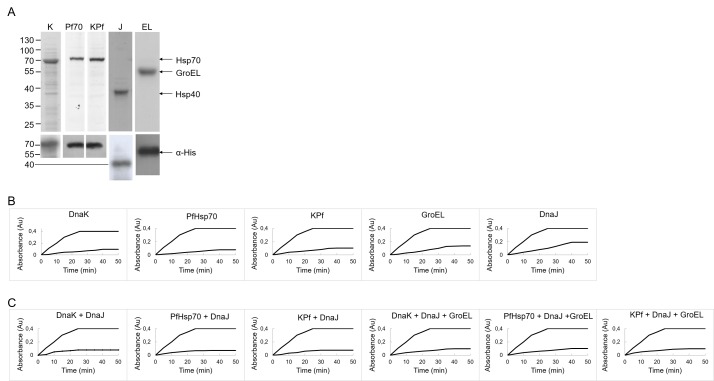
Suppression of heat-induced aggregation of MDH by molecular chaperones. (A) SDS-PAGE analysis confirming the purification of His-tagged DnaK, PfHsp70, KPf, DnaJ and GroEL-GroES overproduced in *E*. *coli* XL1 Blue cells and Western analysis using α-His antibody to detect the proteins. Numbers to the left indicate protein markers (Fermentas) in kDa. Recombinant forms of DnaJ, DnaK, KPf, PfHsp70 and GroEL-GroES at a final concentration of 0.36 μM, either ndependently (B) or in combination (C) were assessed for their ability to inhibit heat-induced aggregation of MDH. The heat induced aggregation of 0.72 μM MDH was monitored at 48°C and 360 nm for 50 mins. The experiment was conducted in the absence or presence of the respective chaperones as indicated.

Having investigated the effects of co-expressing the individual Hsp70 chaperones as well as their combinations with GroEL-GroES on the quality of recombinant PfAdoMetDC, we next set to investigate the function of the chaperones *in vitro*. The *in vitro* function of these chaperones would provide insight on their possible contribution to protein quality control in the cell. Of particular interest to us was to establish the functional compatibility of the plasmodial Hsp70 chaperones and *E*. *coli* chaperones (DnaJ and GroEL). Malate dehydrogenase (MDH) is susceptible to heat stress and for this reason it is widely employed to study the role of heat shock proteins and other molecules with chaperone-like features in protein quality control [28][29][30]. Exposure of MDH to heat stress leads to its aggregation which is detected as increase in turbidity. Some molecular chaperones are known to be capable of suppressing MDH aggregation *in vitro* [29][30][31]. We expressed and purified preparations of the molecular chaperones that were employed in this study as his-tagged species (Fig 8A). As expected, in the absence of molecular chaperones, MDH aggregated upon heat treatment (Fig 8B). The addition of the molecular chaperones (DnaK, PfHsp70, KPf, DnaJ and GroEL) resulted in the suppression of MDH aggregation. DnaJ (by itself) exhibited the lowest activity compared to other chaperones. It is known that DnaJ possesses limited independent chaperone function as its main purpose is to bind mis-folded substrates, handing them over to DnaK for subsequent folding [10]. In the absence of DnaK, DnaJ exhibits limited protein aggregation suppression capability [32]. The *in vitro* chaperone function of PfHsp70 has been previously demonstrated [29]. Although the chaperone activity of KPf has been reported before [13], the findings were based on its ability to protect *E*. *coli dnaK756* cells from heat stress. However, this is the first study that demonstrates that this chimeric Hsp70 chaperone made up of the ATPase domain of DnaK coupled to the substrate binding domain of PfHsp70, KPf, is capable of suppressing protein aggregation *in vitro*.

Overall, the data suggest that Hsp70-DnaJ combinations improved the solubility of MDH *in vitro*. In addition, the introduction of GroEL-GroES to the respective Hsp70-DnaJ combinations did not appear to significantly alter the outcome. Altogether, the functional capabilities exhibited by the chaperones/chaperone combinations whose activities were investigated here may reflect their function in protein quality control when over-expressed in *E*. *coli*. This assay sought to establish the function of the various chaperones in the absence of ATP. Therefore the assay represents only the protein aggregation suppression function of the chaperones and not their capability to refold mis-folded substrates.

## Discussion

It has been proposed that expression of supplemented molecular chaperones could improve the yield and quality of recombinant malarial proteins produced in *E*. *coli* [3][4]. In the current study, first we sought to validate the chaperone role of KPf, the chimeric protein constituted by the ATPase domain of DnaK and the substrate binding domain of PfHsp70. We observed that changes in the arch residues of KPf did not affect its function. However, the V436F mutation representing a substitution in the hydrophobic pocket of KPf abrogated the protein’s function based on a complementation assay. Since introduction of this mutation led to death of *E*. *coli dnaK756* cells subjected to heat stress, this indicates that KPf protected the cells through a specific chaperone function as the V436F mutation is likely to have blocked access of mis-folded protein substrates to the substrate binding cavity of KPf. We therefore surmise that the substrate binding domain of KPf, though of plasmodial origin, is able to recognise mis-folded *E*. *coli* proteins. However, we also hypothesized that KPf would be capable of potentially binding recombinant plasmodial proteins expressed in *E*. *coli*. PfAdoMetDC co-produced in *E*. *coli* with PfHsp70 and its derivative, KPf, demonstrated improved stability and activity compared to the protein that was produced in the presence of supplemented and/or resident *E*. *coli* DnaK. In addition, our findings highlight that supplemented *E*. *coli* DnaK had adverse effects on the quality of PfAdoMetDC both in terms of purity and activity (Figs 4 and 6). *E*. *coli* DnaK binds to its substrates for longer than Hsp70 of eukaryotic origin [19]. For this reason, DnaK may delay the folding process of proteins of eukaryotic origin that are expressed in *E*. *coli*. Since KPf possessed the ATPase domain of DnaK, it may have interacted with GrpE and DnaJ co-chaperones, and this may explain why PfAdoMetDC produced in the presence of KPf possessed a different conformation (Fig 6) and exhibited higher activity (Fig 7) compared to protein co-expressed with PfHsp70. The residues in the ATPase domain of *E*. *coli* DnaK that interact with DnaJ Y145, N147, D148, N170 and T173 and residues G400, D526 and G539 in the peptide binding domain (as reviewed in [8]) are very well conserved in PfHsp70 and by extension KPf (S2 Fig).

Our findings suggest that the Hsp70s of plasmodial origin (KPf and PfHsp70) are primed to interact transiently with PfAdoMetDC to facilitate its proper fold. This is in contrast with DnaK which possibly binds more stably to PfAdoMetDC, leading to the production of a less active, and a more likely inappropriately folded form of the latter. It is also possible that PfHsp70 and its derivative, KPf, may have out-competed the resident DnaK to bind PfAdoMetDC facilitating its improved folding. This is conceivable as both chaperones possess a substrate binding domain that is acquiescent to recognise peptides of plasmodial origin. Alternatively, KPf and PfHsp70 may have acted by indirectly creating protein folding conditions that promoted PfAdoMetDC folding. The contribution of GroEL-GroES towards improved folding of PfAdoMetDC may be due to the possibility that PfAdoMetDC technically qualifies as a GroEL-GroES substrate in spite of its varied species origin. It is known that GroEL-GroES substrates are nearly of the same size as itself; and furthermore GroEL-GroES binds to non-native forms of its substrates but does not bind to their native forms [28][33]. Furthermore, GroEL-GroES binds to mis-folded proteins that are unlikely to be rescued by other molecular chaperones in the cell [34].

It is likely that KPf directly binds PfAdoMetDC to facilitate folding of the latter. In addition, through its possible interaction with DnaJ, KPf may also facilitate refolding of a broad spectrum of *E*. *coli* proteins as well. Interaction of Hsp70 with Hsp40 is crucial to their function in protein folding (foldase function). However, Hsp70 is independently capable of binding mis-folded proteins to stabilise them against aggregation (holdase function) [35][36]. Thus assuming that wild type PfHsp70 may have failed to interact with the DnaK co-chaperones in *E*. *coli*, its role would have been limited to suppressing protein aggregation, amongst them, recombinant PfAdoMetDC. Nonetheless, it is interesting to note that co-expression of either PfHsp70 or KPf with PfAdoMetDC improved the quality of the latter.

The previously reported association of purified recombinant PfAdoMetDC with *E*. *coli* DnaK [7] suggests that the former is produced in a mis-folded state thus may exhibit hydrophobic patches which attract DnaK. In addition, the rate of translation in bacteria is much higher (approximately 20 amino acids per second) than in eukaryotes (approximately 4 amino acids per second) [33]. The slower translation rate in eukaryotes is consistent with the production of multi-domain proteins which require more time for their folding. Therefore the rate of PfAdoMetDC synthesis in *E*. *coli* may have been rapid, giving the protein less time to fold. This would have led to the generation of a product that was not fully folded to which DnaK bound with high affinity. The residence time for DnaK on peptides varies from 30 s to 25 minutes; only proteins that solely depend on DnaK for folding (associated with high frequencies of predicted DnaK binding sites) are released rapidly [37]. On the other hand, proteins that exhibit low cellular abundance, fewer DnaK binding sites and those that tend to assume dynamic structural intermediates (slow folding proteins which do not easily bury their hydrophobic patches) exhibit higher DnaK residence time [37]. Typically such proteins require DnaK for their sustained folding in the cell [37]. Consequently, the extended binding of DnaK to peptides may slow their folding, leading to detrimental consequences. For this reason, it has been proposed that to circumvent DnaK contamination, expression of recombinant proteins in *E*. *coli* Δ*dnaK* cells is recommended [38]. Indeed, our findings suggest that DnaK confounds the folding of PfAdoMetDC. Interestingly, PfAdoMetDC expressed in *E*. *coli* Δ*dnaK* cells displayed enhanced activity. However, the protein was highly susceptible to proteolytic cleavage. This suggests that Hsp70 may not be crucial for PfAdoMetDC production in *E*. *coli*, however it is required for the correct fold and stability of the recombinant protein. In the current study, only PfHsp70 and its derivative, KPf exhibited a positive effect on the quality (improved activity and resilience to proteolytic action) of recombinant PfAdoMetDC produced. It was surprising to us that PfAdoMetDC produced in *E*. *coli* ∆*dnaK* cells exhibited higher activity than protein produced in the presence of DnaK and KPf/PfHsp70. *E*. *coli* ∆*dnaK* cells were cultured at 30°C as this is their ambient growth temperature. This may have resulted in slower rate of protein synthesis and improved quality of recombinant PfAdoMetDC in spite of the absence of DnaK.

Limited proteolysis was used to examine the conformation of PfAdoMetDC expressed both in the absence and presence of supplemented molecular chaperones [39][40]. This technique is based on the hypothesis that the segments of the polypeptide chain that are likely to be accessible to proteinases, are exposed loops within domains or linking segments between domains [40]. It is interesting to note that PfAdoMetDC co-expressed with the various Hsp70 chaperones exhibited the following profile in decreasing order of resilience to proteolytic action; protein co-expressed with: DnaK+DnaJ > KPf+DnaJ > PfHsp70+DnaJ > no supplemented chaperones > ∆*dnaK* (Fig 6A and 6C). On the other hand, PfAdoMetDC produced in the presence of supplemented Hsp70-DnaJ-GroEL-GroES combinations exhibited the following proteolytic stability profiles depending on the chaperone set co-expressed with it: KPf+DnaJ+GroEL-GroES > PfHsp70+DnaJ+GroEL-GroES > DnaK+DnaJ+GroEL-GroES > GroEL-GroES (Fig 7B). Furthermore, the unique proteolytic stability profiles exhibited by PfAdoMetDC produced under the various chaperone conditions testify that slight changes to protein folding conditions in *E*. *coli* have huge implications on the folding fate of recombinant proteins produced in the cell. Overall, the findings suggest that the plasmodial Hsp70s (KPf and its parental protein, PfHsp70) facilitated fold of PfAdoMetDC both in *E*. *coli* cells expressing only resident levels of GroEL-GroES as well as in cells endowed with supplemented GroEL-GroES. On the other hand, supplementing both DnaK and GroEL-GroES did not improve the resistance of PfAdoMetDC to proteolytic cleavage compared to supplementing with DnaK only.

We further demonstrated the chaperone activity of KPf *in vitro* by showing its capability to suppress heat-induced aggregation of MDH, an aggregation prone protein. This is the first study showing the independent chaperone capability of this chimeric Hsp70 protein. We surmise that KPf similarly bound to recalcitrant PfAdoMetDC recombinant protein produced in *E*. *coli*, suppressing its aggregation. The possible interaction of KPf with *E*. *coli* DnaK chaperones, such as GrpE and DnaJ makes it possible for KPf to facilitate refolding of PfAdoMetDC co-expressed with it in *E*. *coli*. This is because DnaJ speeds up the otherwise rate-limiting ATP hydrolysis step of DnaK binding, while GrpE facilitates nucleotide exchange.

In eukaryotes, Hsp70 mediates substrate transfer to Hsp60 (TRiC for TCP-1 ring complex) by directly interacting with TRiC [41]. However, TRiC does not share the same specificity for substrates as GroEL; substrate sequence and structure varies and may even reach 100–120 kDa in size [42]. In addition, TRiC is more intimately linked to Hsp70 in eukaryotes, facilitating direct substrate transfer from Hsp70 to TRiC [43]. The TRiC protein folding cycle occurs at a much slower rate compared to that of GroEL-GroES and thus more time is provided for encapsulation and folding of substrates by the chaperonin [34]. The differences between the cooperation of Hsp70 and TRiC in eukaryotes compared to the DnaK+GroEL-GroES functional partnership may explain why certain proteins of eukaryotic origin may not fold properly in *E*. *coli* [44]. It is possible that KPf may cooperate with GroEL-GroES more productively to facilitate processing of recombinant malarial proteins produced in *E*. *coli* compared to the DnaK-GroEL-GroES partnership. However, this remains to be directly validated.

We hypothesize that because of their acquiescence to recognising PfAdoMetDC, PfHsp70 and KPf took over the responsibility of facilitating PfAdoMetDC from resident DnaK. The expression of plasmodial Hsp70s (KPf and PfHsp70) in the presence of supplemented GroEL-GroES led to the recovery of purer PfAdoMetDC recombinant protein. Based on size criteria and its inclination to being recalcitrant, PfAdoMetDC is most likely a substrate of both Hsp70 and GroEL-GroES. GroEL-GroES is known to rescue the folding of proteins that other *E*. *coli* chaperones do not fold effectively and most of its substrates are nearly the same size as itself [33][34]. PfAdoMetDC fits both criteria and may therefore require GroEL-GroES to facilitate its full processing. It is plausible that KPf provides an ideal Hsp70 to partner with GroEL-GroES in facilitating the fold of PfAdoMetDC. It remains to be studied however, if the role of KPf could be extended to facilitate processing of other recalcitrant malarial recombinant proteins.

## Materials and Methods

### *E*. *coli* strains and plasmids

The *E*. *coli* ∆*dnaK* strain, BB1553 (MC4100 ∆*dnaK52*::*CmR sidB1*) and *E*. *coli dnaK* mutant strain, BB2362 (*dnaK756 recA*::*Tc*^*R*^ *pDMI*,*1*) and plasmids pBB535, expressing genes DnaK and DnaJ, and pBB542, expressing DnaK, DnaJ and GroEL-GroES, were a kind donation from Dr Bernd Bukau (Heidelberg University, Germany). *E*. *coli dnaK756* BB2362 strain is resistant to bacteriophage lambda [45], and is unable to grow above 40°C [45][46]. BB2362 expresses mutant DnaK with three glycine-to-aspartate substitutions [47]. Both plasmids pBB535 and pBB542 are under the control of the IPTG regulated promoter PA1/lacO-1 and carries spectinomycin resistance [45]. We have routinely used the construct pQE30/PfHsp70 to express PfHsp70 in *E*. *coli* [13][29]. The pASK-IBA3/PfAdoMetDC hosting the codon-harmonised *PfAdoMetDC* gene encoding for the α-subunit of the protein (approximately 60 kDa) has previously been described [7]. The PfAdoMetDC was expressed as a C-terminally Strep-tagged molecule. The pASK-IBA3 plasmid is under the control of the tet promoter which is regulated by anhydrotetracycline (AHT). The tet repressor keeps the promoter in a repressed state until the addition of AHT; expression leakage is thus minimal. A description of all strains and plasmids used in this study is provided in supporting information (S1 Table and S2 Table).

### Introduction of arch and hydrophobic pocket substitutions in KPf

To determine the role of the arch and hydrophobic pocket residues of the substrate binding cavity of KPf, mutations were introduced in this subdomain based on the same approach and primers that we previously employed to introduce similar changes on the full length PfHsp70 protein [29]. In the current study, we made the changes on KPf, a derivative of PfHsp70 that possessed an ATPase domain from DnaK. Plasmid pQE60/KPf was used as the parental DNA to generate modified plasmids encoding KPf with mutations in the substrate binding cavities. The Stratagene QuikChange site-directed mutagenesis kit was used to modify the plasmids, following the instructions of the supplier. The following are the derivatives we sought to generate from a construct, pQE60/KPf [13]: pQE60/KPf: pQE60/KPf-A404Y (encoding for KPf-A404Y protein), pQE60/KPf-Y429A (encoding for KPf-Y429A protein), and pQE60/KPf-A436F (encoding for KPf-A436F protein). All the changes were verified by DNA sequencing.

### Investigating the role of the arch and hydrophobic residues of KPf using a complementation assay

We previously demonstrated that PfHsp70 and KPf both confer cytoprotection to *E*. *coli dnaK756* against heat stress [13]. In the current study, we introduced changes to the residues in the substrate binding cavity and hydrophobic pocket of KPf. Our aim was to validate if the cytoprotective function of KPf in *E*. *coli* is dependent on the integrity of residues constituting the arch and hydrophobic pockets that are located in its C-terminal substrate binding domain. *E*. *coli dnaK756* cells were transformed with plasmids encoding the proteins KPf and its respective derivatives with mutations in the substrate binding cavities. pQE60 plasmid vector was used as a negative control. Cells transformed with pQE60/DnaK constituted a positive control. *E*. *coli dnaK756* is resistant to bacteriophage lambda [46], and is unable to grow above 40°C [46][47]. This strain’s resident DnaK contains three amino acid substitutions, one of which reduces its affinity for GrpE, whilst the two other substitutions elevate the basal ATPase activity of DnaK [48]. The cells were transformed using plasmids encoding the KPf variants before being subjected to heat stress (43.5°C) in order to assess the capabilities of the respective proteins to reverse the thermo-sensitivity of the cells. Freshly transformed *E*. *coli dnaK756* cells were grown overnight at 30°C in 2 x YT broth (16 g of tryptone powder, 10 g of yeast extract powder and 5 g of sodium chloride in 1000 mL of double distilled water) containing 50 μg/ml kanamycin, 10 μg/ml tetracycline and 100 μg/ml ampicillin. The overnight inoculum was transferred into fresh broth and incubated under the same growth conditions. At mid-log phase of growth, some cells were induced with 1 mM IPTG whilst others were not. The cells were left to grow to A_600_ = 2.0. The cultures were standardised to a cell density of 0.2 A_600_ before being spotted onto 2 x YT agar plates containing the necessary antibiotics and 20 μM IPTG, and incubated overnight at 37°C and 43.5°C, respectively.

### Construction of expression vectors pQE30-DnaJ, pQE30-KPf and pQE30-GroEL-GroES

DNA segments encoding DnaJ, KPf and GroEL-GroES were PCR amplified from pBB535 and cloned into pQE30 (Qiagen, Germany) plasmid vector in frame with an N-terminal His-tag to facilitate their purification using nickel affinity chromatography. pQE30-DnaJ was constructed using the forward primer 5´-ATCACGGATCCATGGTCTAAGCAAGATATTATTACG-3´) with a *Bam*HI restriction site and reverse primer (5’-TTGGCTGCAGTTAGCGGGTCAGGTCG-3´) with a *Pst*I restriction site. The KPf encoding segment was PCR amplified from pQE60-KPf, previously developed for the over-expression of the protein [13]. Forward (5´-ATCACGGATCCATGGTGAAACTCTGG-3´) and reverse (5´-TAATTAAGCTTTTCCACTTGGCATTCC-3´) primers containing *Bam*HI and *Hind*III restriction sites (underlined), respectively, were used for the construction of pQE30-KPf. The GroEL-GroES encoding segment was PCR amplified using forward primer 5´-TCCGCATGCATGGCACTAAAGAC-3´ containing restriction site *Sph*I (underlined) and reverse primer 5´-TAATTAAGCTTTTACATCATGCCGCCC-3´ containing *Hind*III (underlined). The integrity of the resultant His-tagged constructs (pQE30-DnaJ, pQE30-KPf and pQE30-GroEL-GroES) was confirmed by restriction analysis and DNA sequencing.

### Production of pBB535 and pBB542 based constructs for the expression of PfHp70 and its derivative

To facilitate co-expression of PfHsp70 and its derivative, KPf, with PfAdoMetDC in *E*. *coli*, plasmid vectors were selected based on compatible origins of replication and independent antibiotic selection. pBB535, originally encoding for DnaK and DnaJ, was altered to encode for PfHsp70+DnaJ, and KPf+DnaJ, respectively. Similarly, pBB542, encoding for DnaK, DnaJ and GroEL-GroES, was modified such that *DnaK* was replaced by *PfHsp70* and *KPf*, respectively.

The pBB535 construct was used as template to conduct site-directed mutagenesis to generate the constructs pBB535-PfHsp70 and pBB535-KPf, respectively. A *Bam*HI was introduced before the starting codon of DnaK followed by *Sma*I site that was introduced after the stop codon of DnaK. The forward primer, 5´-GACTCTCTTCCGGGGATCCATGCCATACCGCGAAAGGTTTTGC-3´ and reverse primer, 5’-GCAAAACCTTTCGCGGTATGGCATGGATCCCCGGAAGAGAGTC-3’ were used to introduce the *Bam*HI site, respectively. The introduction of the *Sma*I was facilitated using the forward primer, 5´-CAAAGACAAAAAATAACCCGGGATAAACGGGTAATTATACTGACACGGGC-3´; and reverse primer, 5´-GCCCGTGTCAGTATAATTACCCGTTTATCCCGGGTTATTTTTTGTCTTTG-3´, respectively.

For the generation of constructs expressing GroEL-GroES (pBB542-PfHsp70/GroEL-GroES and pBB542-KPf/GroEL-GroES), the pBB542 construct was used as template. PfHsp70 and KPf encoding segments were amplified by the polymerase chain reaction (PCR) from pQE30-PfHsp70 and pQE60-KPf [13][29][49], respectively. To amplify *PfHsp70*, the forward primer, 5´-GGATCCATGGCTAGTGCAAAAGGTTCAAACC-3´ and reverse primer, 5´-GGGCCCTTAATCAACTTCTTCAAC-3´ were employed. KPf amplification was conducted using the forward primer, 5′-GGATCCATGGGTAAAATAATTGGTATCGAC-3′) and reverse primer, 5´-GGGCCCTTAATCAACTTCTTCAAC-3´. The primers contained *Bam*HI and *Sma*I restriction sites (underlined), respectively. The PCR products were then restricted and inserted between *Bam*HI and *Sma*I restriction site in pBB542 plasmid vector. The integrity of the resultant constructs was confirmed by restriction analysis and DNA sequencing.

### Expression and purification of recombinant proteins

An overnight culture was prepared by inoculating *E*. *coli* BL21 (DE3) Star [pASK-IBA3/PfAdoMetDC] into 5 ml LB broth supplemented with 100 μg/ml ampicillin and grown at 37°C with shaking. The overnight culture was diluted 1/100 and allowed to grow at 37°C until the optical density (OD_600nm_) reached 0.5. At this stage, protein induction was initiated using 2 ng/ml anhydrotetracycline (AHT) (IBA GmbH, Germany). Samples were withdrawn at various stages of growth to monitor the expression of PfAdoMetDC in the absence of supplemented chaperones. Co-expression of PfAdoMetDC with the chaperone sets DnaK+DnaJ (K); KPf+DnaJ (KPf); or PfHp70+DnaJ (Pf70) entailed co-transformation of *E*. *coli* BL21 (DE3) Star with the constructs expressing PfAdoMetDC and either pBB535; pBB535-KPf; or pBB535-PfHsp70, respectively. Similarly, co-expression of PfAdoMetDC with the chaperones sets DnaK+DnaJ+GroEL-GroES (K-EL); KPf+DnaJ+GroEL-GroES (KPf-EL); or PfHp70+DnaJ+GroEL-GroES (Pf70-EL) entailed co-transformation of *E*. *coli* BL21 (DE3) Star cells with the constructs expressing PfAdoMetDC and either pBB542; pBB542-KPf/GroEL-GroES; or pBB542-PfHsp70/GroEL-GroES, respectively. The expression of chaperones was initiated first by the addition of 1 mM IPTG at OD_600nm_ 0.2 followed by the induction of PfAdoMetDC at an OD_600nm_ of 0.7 using 2 ng/ml AHT.

*E*. *coli* BB1553 (MC4100 Δ*dnaK52*::CmR sidB1) cells lack the *dnaK* gene [50]. We investigated the expression of PfAdoMetDC in these cells. Briefly, competent *E*. *coli ∆dnaK* cells were transformed with the pASK-IBA3/PfAdoMetDC construct. Following transformation, a single colony was inoculated into 2 x YT broth (16 g of tryptone powder, 10 g of yeast extract powder and 5 g of sodium chloride in 1000 mL of double distilled water) supplemented with 35 μgmL-1 chloramphenicol and 100 μgmL-1 ampicillin and the culture was left to grow overnight at 30°C. The following morning, 5 μL of inoculum from the overnight culture was transferred into 45 mL of fresh 2 x YT broth, supplemented with 35 μgmL-1 chloramphenicol and 100 μg/mL ampicillin. The cells were incubated at 30°C with shaking to optical density (OD600) of 0.6. PfAdoMetDC production was induced by the addition of 2 ng/ml AHT. The cells were harvested for protein expression and purification studies.

Purification of PfAdoMetDC was conducted by using the Strep-Tactin Sepharose (Invitrogen) column purification system as described previously [7][27]. Similarly, the his-tagged proteins were expressed in *E*. *coli* XL1 Blue off their respective constructs: pBB46/pQE60-DnaK, pQE30-PfHsp70, pQE30-KPf, pQE30-DnaJ and pQE30-GroEL-GroES. The proteins were purified as previously described [29][51] using the HisPur Ni-NTA resin purification system (Pierce, USA). Purified protein was quantified using the Bradford assay [52]. Protein expression, solubility and purification were confirmed using SDS-PAGE analysis. Western analysis was used to verify the identity of said proteins using monoclonal α-Strep-tag II antibodies (Novagen), α-PfHsp70 antibodies [53], α-DnaK antibodies (Abcam) and monoclonal mouse α-his antibodies (Pierce, USA).

### Analysis of PfAdoMetDC using limited proteolysis

We sought to investigate conformational changes of PfAdoMetDC that was expressed in *E*. *coli* BL21 (DE3) Star cells in the absence and presence of supplemented molecular chaperones using limited proteolysis [41]. We also subjected PfAdoMetDC protein that was expressed and purified from *E*. *coli ∆dnaK* cells to limited proteolytic analysis. Purified PfAdoMetDC (0.2 mg/ml) was incubated with 0.33 mg/ml proteinase K at 37°C for 30 minutes. Proteolytic digestion of PfAdoMetDC was analysed using SDS-PAGE and Western analysis was for verification using monoclonal α-Strep-tag II antibodies to detect fragments recombinantly produced with the C-terminal located Strep-tag II.

### Assessment of the enzymatic activity of PfAdoMetDC

The enzymatic activity of PfAdoMetDC preparations that had been expressed under varied protein folding conditions was determined. The assay constituents included 5 ug enzyme, 100 uM S-adenosy-L-methionine chloride (Sigma-Aldrich, Germany) and 50 nCi S-[Carboxyl-14 C] adenosyl-L-methionine (55 mCi/mmol, Amersham Biosciences, England) in assay buffer (50 mM KH_2_PO4 pH 7.5, 1 mM EDTA, 1 mM DTT) as previously described [7][54]. All the assays were performed in triplicate and the specific enzyme activities were expressed as the amount of CO_2_ produced in nmol/min/mg.

### Assessment of the effectiveness of molecular chaperones to suppress protein aggregation *in vitro*

The ability of the recombinant Hsp70 proteins (PfHsp70, KPf and DnaK) to suppress heat-induced aggregation of malate dehydrogenase (MDH) was determined spectrophotometrically based on a previously reported assay [29][30]. Furthermore, the heat-induced aggregation of MDH was investigated in the presence of DnaJ in a ratio of 2:1 (DnaJ:Hsp70) and GroEL-GroES in a ratio of 1:1 (GroEL-GroES:Hsp70). The proteins were suspended in assay buffer (100 mM NaCl, 50 mM Tris, pH 7.4). The aggregation of the protein was determined by reading absorbance at 360 nm using a 96-well plate reader (BioteK, ELx808). A non-chaperone, BSA, was used as a control.

## Supporting Information

S1 Fig KPf and PfHsp70 do not co-purify with PfAdoMetDC. Western blot representing the purification of PfAdoMetDC expressed in *E*. *coli* BL21 (DE3) Star cells rehosted with various chaperone combinations. Lanes: **U**–PfAdoMetDC expressed in the absence of supplemented chaperones; K–PfAdoMetDC co-expressed with supplemented DnaK; KPf–PfAdoMetDC expressed in cells supplemented with KPf; Pf70 –PfAdoMetDC expressed in cells supplemented with PfHsp70; K-EL–PfAdoMetDC expressed in cells supplemented with DnaK and GroEL-GroES; KP-EL–PfAdoMetDC expressed in cells supplemented with KPf and GroEL-GroES; Pf70-EL–PfAdoMetDC expressed in cells supplemented with PfHsp70 and GroEL-GroES; +C–positive consisting of purified PfHsp70 protein. Western blot analysis of PfHsp70 (70 kDa) detected using α-PfHsp70 antibody. Numbers to the left represent protein markers (Fermentas) in kDa.
(TIF)

S2 Fig Sequence alignment of PfHsp70 and *E*. *coli* DnaK. Sequence alignment of *E*. *coli* DnaK (accession number: BAA01595.1) and PfHsp70 (accession number: PF08_0054) were conducted using ClustalW and Boxshade. The following structural features are highlighted: the highly conserved linker segment (black horizontal line) which separates the ATPase domain from the peptide binding domain. Residues Y145, N147, D148, N170 and T173 in the ATPase domain that interact with DnaJ as reviewed by Shonhai et al (8) are shown with black arrows. Residues G400, D526 and G539 in the peptide binding domain of DnaK that are important for interaction with DnaJ, and the aligned residues in PfHsp70 are shown as black arrows. Identical residues are presented in white against a black background and similar residues are shown in black against a grey background).
(TIF)

S1 Table *E*. *coli* strains and plasmids used in this study. (DOCX)

S2 Table Description of primers used towards generation of destination plasmids. (DOCX)
