# Supplementary figures and images for: Use of a Chimeric Hsp70 to Enhance the Quality of Recombinant Plasmodium falciparum S-Adenosylmethionine Decarboxylase Protein Produced in Escherichia coli
Source: PLoS One. 2016 Mar 31;11(3):e0152626. doi: 10.1371/journal.pone.0152626 (PMC4816425; doi:10.1371/journal.pone.0152626)

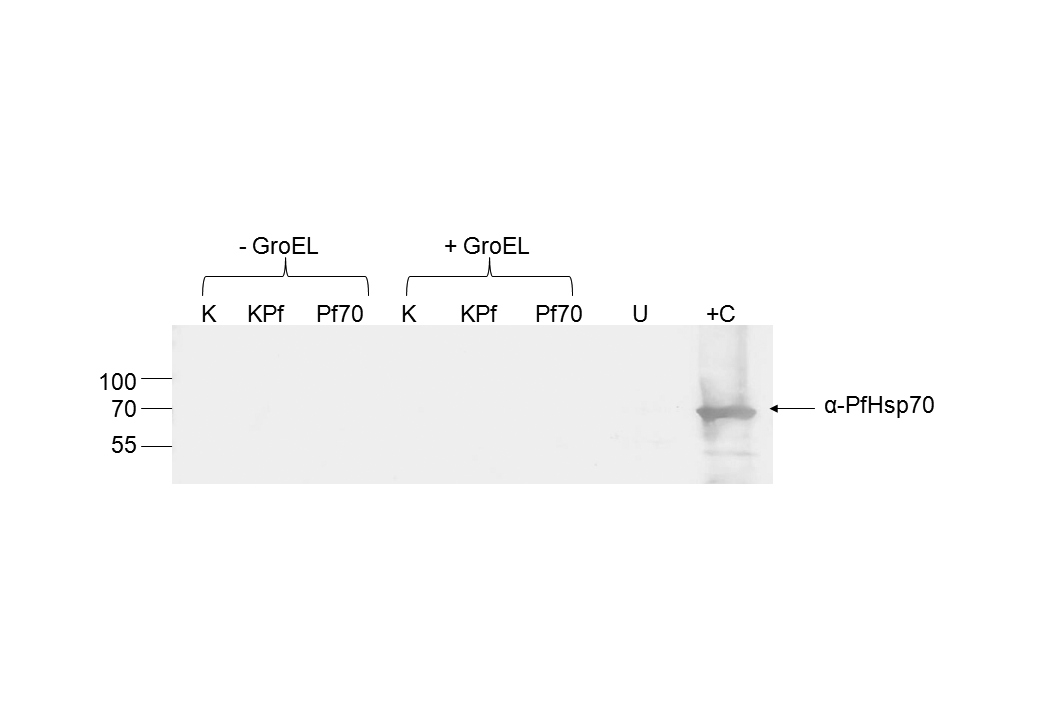

Supplement: S1 Fig — Western blot representing the purification of PfAdoMetDC expressed in E. coli BL21 (DE3) Star cells rehosted with various chaperone combinations. Lanes: U–PfAdoMetDC expressed in the absence of supplemented chaperones; K–PfAdoMetDC co-expressed with supplemented DnaK; KPf–PfAdoMetDC expressed in cells supplemented with KPf; Pf70 –PfAdoMetDC expressed in cells supplemented with PfHsp70; K-EL–PfAdoMetDC expressed in cells supplemented with DnaK and GroEL-GroES; KP-EL–PfAdoMetDC expressed in cells supplemented with KPf and GroEL-GroES; Pf70-EL–PfAdoMetDC expressed in cells supplemented with PfHsp70 and GroEL-GroES; +C–positive consisting of purified PfHsp70 protein. Western blot analysis of PfHsp70 (70 kDa) detected using α-PfHsp70 antibody. Numbers to the left represent protein markers (Fermentas) in kDa. (TIF) [file pone.0152626.s001.tif]

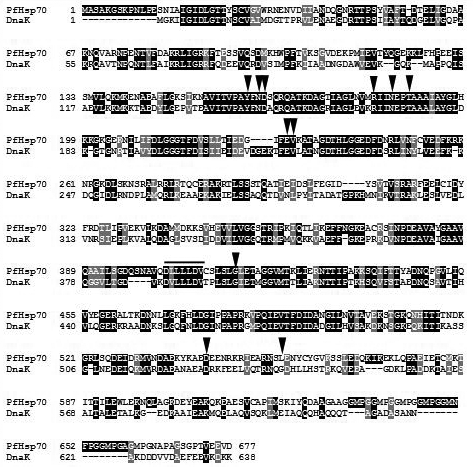

Supplement: S2 Fig — Sequence alignment of E. coli DnaK (accession number: BAA01595.1) and PfHsp70 (accession number: PF08_0054) were conducted using ClustalW and Boxshade. The following structural features are highlighted: the highly conserved linker segment (black horizontal line) which separates the ATPase domain from the peptide binding domain. Residues Y145, N147, D148, N170 and T173 in the ATPase domain that interact with DnaJ as reviewed by Shonhai et al (8) are shown with black arrows. Residues G400, D526 and G539 in the peptide binding domain of DnaK that are important for interaction with DnaJ, and the aligned residues in PfHsp70 are shown as black arrows. Identical residues are presented in white against a black background and similar residues are shown in black against a grey background). (TIF) [file pone.0152626.s002.tif]
