# Supplementary material for: Use of a Chimeric Hsp70 to Enhance the Quality of Recombinant Plasmodium falciparum S-Adenosylmethionine Decarboxylase Protein Produced in Escherichia coli
Source: PLoS One. 2016 Mar 31;11(3):e0152626. doi: 10.1371/journal.pone.0152626 (PMC4816425; doi:10.1371/journal.pone.0152626)
Supplement: S1 Table — (DOCX) [file pone.0152626.s003.docx]

**S1 Table. *E. coli* strains and plasmids used in this study**

| **Strains and plasmids** | **Description** | **Source/Reference** |
| --- | --- | --- |
| ***E. coli* strains** | | |
| BB1553 | MC4100 ∆*dnaK52::Cm^R^ sidB1* | [36][53] |
| BB2362 | *dnaK756 recA::Tc^R^ pDMI,1* | [46] |
| BL21 Star^TM^ (DE3) cells | F^-^*omp*T *hsd*S_B_ (r_B_^-^, m_B_^-^) *galdcmrne*131 (DE3) |  |
| XL1 Blue | *recA1 endA1 gyrA96 thi-1 hsdR17 supE44 relA1 lac [F´ proAB lacIqZM15 Tn10* (Tetr)]. | Bullock et al (1987) |
| **Plasmids** | | |
| pQE30-DnaJ | pQE30 encoding *E. coli DnaJ*, Amp^R^ | This study |
| pQE30-KPf | pQE30 encoding *KPf*, Amp^R^ | This study |
| pQE30-GroEL-GroES | pQE30 encoding *E. coli GroEL-GroES*, Amp^R^ | This study |
| pQE30/PfHsp70 | pQE30 encoding *PfHsp70*, Amp^R^ | [13][34] |
| pASK-IBA3/PfAdoMetDC | pASK-IBA3 encoding codon-harmonized *PfAdoMetDC*, Amp^R^ | [7] |
| pQE60/KPf | pQE60 encoding *KPf*, Amp^R^ | [13] |
| pQE60/KPf-A404Y | pQE60 encoding *KPf*-A404Y, Amp^R^ | This study |
| pQE60/KPf-Y429A | pQE60 encoding *KPf*-Y429A, Amp^R^ | This study |
| pQE60/KPf-A436F | pQE60 encoding *KPf*-A436F, Amp^R^ | This study |
| pBB46/pQE60-DnaK | pQE60 encoding *E. coli DnaK*, Amp^R^ | Burkholder et al (1994) |
| pBB535 | pBB535 encoding *E. coli DnaK* and *DnaJ*, Amp^R^ | [41] |
| pBB535-PfHsp70 | pBB535 encoding *PfHsp70* and *E. coli DnaJ*, Amp^R^ | This study |
| pBB535-KPf | pBB535 encoding *KPf* and *E. coli DnaJ*, Amp^R^ | This study |
| pBB542 | pBB542 encoding *E. coli DnaK, DnaJ* and *GroEL-GroES*, Amp^R^ | [41] |
| pBB542-PfHsp70/GroEL-GroES | pBB542 encoding *PfHsp70* and *E. coli DnaJ* and *GroEL-GroES*, Amp^R^ | This study |
| pBB542-KPf/GroEL-GroES | pBB542 encoding *KPf* and *E. coli DnaJ* and *GroEL-GroES*, Amp^R^ | This study |

Additional References

-Bullock WO, Fernandez JM, Short JM. XL1 Blue: A high efficiency plasmid transforming *recA* *Escherichia coli* strain with beta-galactosidase selection. Biotechniques. 1987, 5:376-378.

-Burkholder WF, Panagiotidis CA, Silverstein SJ, Cegielska A, Gottesman ME, Gaitanaris GA. Isolation and characterization of an *Escherichia coli* DnaK mutant with impaired ATPase activity. J Mol Biol. 1994, 242:364-377.
