# Supplementary material for: Use of a Chimeric Hsp70 to Enhance the Quality of Recombinant Plasmodium falciparum S-Adenosylmethionine Decarboxylase Protein Produced in Escherichia coli
Source: PLoS One. 2016 Mar 31;11(3):e0152626. doi: 10.1371/journal.pone.0152626 (PMC4816425; doi:10.1371/journal.pone.0152626)
Supplement: S2 Table — (DOCX) [file pone.0152626.s004.docx]

**S2 Table. Description of primers used towards generation of destination plasmids**

| **Primer** | **Primer length (5’ to 3’)** | **Restriction enzyme** | **Destination plasmid** |
| --- | --- | --- | --- |
| **pQE30-based constructs** | | | |
| 1F | ATCACGGATCCATGGTCTAAGCAAGATATTATTACG | *Bam*HI | pQE30-DnaJ |
| 1R | TTGGCTGCAGTTAGCGGGTCAGGTCG | *Pst*I |  |
| 2F | ATCACGGATCCATGGTGAAACTCTGG | *Bam*HI | pQE30-KPf |
| 2R | TAATTAAGCTTTTCCACTTGGCATTCC | *Hind*III |  |
| 3F | TCCGCATGCATGGCACTAAAGAC | *Sph*I | pQE30-GroEL-GroES |
| 3R | TAATTAAGCTTTTACATCATGCCGCCC | *Hind*III |  |
| **pBB535-based constructs** | | | |
| 4F | GACTCTCTTCCGGGGATCCATGCCATACCGCGAAAGGTTTTGC | *Bam*HI | pBB535-PfHsp70/DnaJ |
| 4R | CAAAGACAAAAAATAACCCGGGATAAACGGGTAATTATACTGACACGGGC | *Sma*I |  |
| 5F | GCAAAACCTTTCGCGGTATGGCATGGATCCCCGGAAGAGAGTC | *Bam*HI | pBB535-KPf/DnaJ |
| 5R | GCCCGTGTCAGTATAATTACCCGTTTATCCCGGGTTATTTTTTGTCTTTG | *Sma*I |  |
| **pBB542-based constructs** | | | |
| 4F | GACTCTCTTCCGGGGATCCATGCCATACCGCGAAAGGTTTTGC | *Bam*HI | pBB542-PfHsp70/GroEL/DnaJ |
| 4R | CAAAGACAAAAAATAACCCGGGATAAACGGGTAATTATACTGACACGGGC | *Sma*I |  |
| 5F | GCAAAACCTTTCGCGGTATGGCATGGATCCCCGGAAGAGAGTC | *Bam*HI | pBB542-KPf/GroEL/DnaJ |
| 5R | GCCCGTGTCAGTATAATTACCCGTTTATCCCGGGTTATTTTTTGTCTTTG | *Sma*I |  |
